# Automated reference tissue normalization of T2-weighted MR images of the prostate using object recognition

**DOI:** 10.1007/s10334-020-00871-3

**Published:** 2020-07-31

**Authors:** Mohammed R. S. Sunoqrot, Gabriel A. Nketiah, Kirsten M. Selnæs, Tone F. Bathen, Mattijs Elschot

**Affiliations:** 1 Department of Circulation and Medical Imaging, NTNU, Norwegian University of Science and Technology, 7030 Trondheim, Norway; 2 Department of Radiology and Nuclear Medicine, St. Olavs Hospital, Trondheim University Hospital, 7030 Trondheim, Norway

**Keywords:** Prostate, Reference tissue, Normalization, MRI, Object recognition

## Abstract

**Objectives:**

To develop and evaluate an automated method for prostate T2-weighted (T2W) image normalization using dual-reference (fat and muscle) tissue.

**Materials and methods:**

Transverse T2W images from the publicly available PROMISE12 (*N* = 80) and PROSTATEx (*N* = 202) challenge datasets, and an in-house collected dataset (*N* = 60) were used. Aggregate channel features object detectors were trained to detect reference fat and muscle tissue regions, which were processed and utilized to normalize the 3D images by linear scaling. Mean prostate pseudo T2 values after normalization were compared to literature values. Inter-patient histogram intersections of voxel intensities in the prostate were compared between our approach, the original images, and other commonly used normalization methods. Healthy vs. malignant tissue classification performance was compared before and after normalization.

**Results:**

The prostate pseudo T2 values of the three tested datasets (mean ± standard deviation = 78.49 ± 9.42, 79.69 ± 6.34 and 79.29 ± 6.30 ms) corresponded well to T2 values from literature (80 ± 34 ms). Our normalization approach resulted in significantly higher (*p* < 0.001) inter-patient histogram intersections (median = 0.746) than the original images (median = 0.417) and most other normalization methods. Healthy vs. malignant classification also improved significantly (*p* < 0.001) in peripheral (AUC 0.826 vs. 0.769) and transition (AUC 0.743 vs. 0.678) zones.

**Conclusion:**

An automated dual-reference tissue normalization of T2W images could help improve the quantitative assessment of prostate cancer.

**Electronic supplementary material:**

The online version of this article (10.1007/s10334-020-00871-3) contains supplementary material, which is available to authorized users.

## Introduction

Prostate cancer is the second most commonly diagnosed cancer and the leading cause of cancer-related deaths among men worldwide [1]. Multiparametric magnetic resonance imaging (mpMRI) has been established as a valuable diagnostic tool for prostate cancer [2, 3]. T2-weighted (T2W) MR imaging is considered an essential pillar of mpMRI for prostate cancer diagnosis due to the high spatial resolution and the superior anatomical details it provides [3–5]. However, unlike other mpMRI sequences such as diffusion-weighted and dynamic contrast-enhanced imaging, the use of T2W imaging has mainly been limited to a qualitative evaluation of prostate anomalies. Its utility for quantitative analysis is hindered by, among other things, non-standard signal intensity (SI) attributed to scanner parameters such as the field strength, coil type, signal amplification, and acquisition protocols [6–9]. To make use of T2W images for quantitative analysis, an image processing step called SI normalization is often required, which theoretically removes the variation in SI between images from different scan sessions. Consequently, SI normalization enables comparing T2W image values from different patients (inter-patient comparison), patient follow-up at multiple scans over time (intra-patient comparison), and tissue classification tasks in the setting of a radiomics or computer-assisted diagnosis approach [10, 11].

SI normalization is not new, and over the years, different approaches have been proposed for prostate imaging. Due to their simplicity, histogram-based approaches, which typically depend on pre-set histogram landmarks to deform or rescale the SI [7, 12], have become the most commonly used [10, 13–16]. A drawback of these methods is that they usually rely on the content in the complete 2D or 3D image, which is subject to variation due to differences in scan settings (e.g. the field-of-view) and patient-related factors (e.g. bladder filling). Recently, SI normalization utilizing single or multiple reference tissues has shown promise as an alternative to histogram-based methods [17–21]. In single reference tissue normalization, the original T2W image SI is scaled by the SI in the corresponding reference tissue region-of-interest (ROI). One common example of this in the prostate is normalization to the SI of the obturator internus or levator ani muscles [17, 22–24]. Multi-reference tissue normalization, on the other hand, utilizes the SIs of multiple reference tissues to create a linear or non-linear regression model to estimate the normalized T2W image values [18, 19]. The assumption is that reference tissue-based normalization is less sensitive to variations in scan settings and patient-related factors. However, a key aspect of this approach is labelling the reference tissues, to enable SI extraction. Currently, this is done manually, which is a time-consuming and tedious process. Automated delineation of reference tissue ROIs would make the approach more efficient and could possibly facilitate its integration into clinical practice. This can for example be achieved using automated semantic segmentation or object detection methods. In comparison with semantic segmentation, object detection requires less processing power, time and data [25, 26].

The contribution of this work is a novel method for automated dual-reference tissue normalization of T2W images of the prostate, based on object recognition to extract the reference tissue ROIs. We compared the automatically extracted reference tissue intensities with those of manually delineated ROIs, and evaluated the merit of the proposed method for inter- and intra-patient comparison of T2W image intensities and for the classification of malignant lesions versus healthy prostate tissue.

## Materials and methods

### Datasets

In this study, transverse T2W images from three separate datasets were used: the PROMISE12 grand challenge dataset (*N* = 80) [27], the PROSTATEx challenge dataset (*N* = 202) [28] and a dataset of in-house collected T2W images from patients who underwent two sequential MRI scans for detection and biopsy-guiding, respectively (*N* = 60). The Regional Committee for Medical and Health Research Ethics (REC Mid Norway) approved the use of the in-house collected dataset (identifier 2017/576) and granted permission for passive consent to be used, whereas the two other datasets were publicly available.

The PROMISE12 dataset [27] consists of multi-centre and multi-vendor transverse T2W images obtained with different field strengths, acquisition protocols and coils. It also includes manual expert segmentations of the whole prostate for 50 cases. The PROSTATEx challenge dataset [28] consists of pre-biopsy mpMRI sequences acquired at Radboud University Medical Centre, Nijmegen, Netherlands. The whole prostate, peripheral zone, and cancer-suspicious volumes of interest (VOIs) were manually delineated by radiologists (at Miller School of Medicine, Miami, FL, USA) based on targeted biopsy locations provided by the challenge organizers. The presence of clinically significant prostate cancer (Gleason score > 3 + 3) in the targeted biopsy cores was then used to label each cancer-suspicious VOI as a true positive (malignant) or false positive radiological finding. The rest of the prostate was considered healthy tissue.

The in-house collected dataset was obtained from St. Olavs Hospital, Trondheim University Hospital, Trondheim, Norway between March 2015 and December 2017. It consists of pairs of pre-biopsy 3 T images from 60 patients (median age = 65.5 years; range 47–75 years) acquired at two different time points: first, at the initial visit for detection of prostate cancer (scan 1), and second, during an MR-guided biopsy procedure (scan 2). The interval between scans ranged 1–71 days with a median interval of 7 days. T2W imaging was performed on a Magnetom Skyra 3 T MRI system (Siemens, Erlangen, Germany) with a turbo spin-echo sequence (Scan 1: repetition time/echo time = 4800–9520/104 ms, 320 × 320 – 384 × 384 matrix size, 26–32 slices, 3 mm slice thickness and 0.5 × 0.5–0.6 × 0.6 mm^2^ in plane resolution. Scan 2: repetition time/echo time = 5660–7740/101–104 ms, 320 × 320–384 × 384 matrix size, 19–26 slices, 3 mm slice thickness and 0.5 × 0.5–0.6 × 0.6 mm^2^ in plane resolution). The whole prostate volumes were manually delineated by a radiologist in training.

### Proposed intensity normalization method

**Fig. 1:**
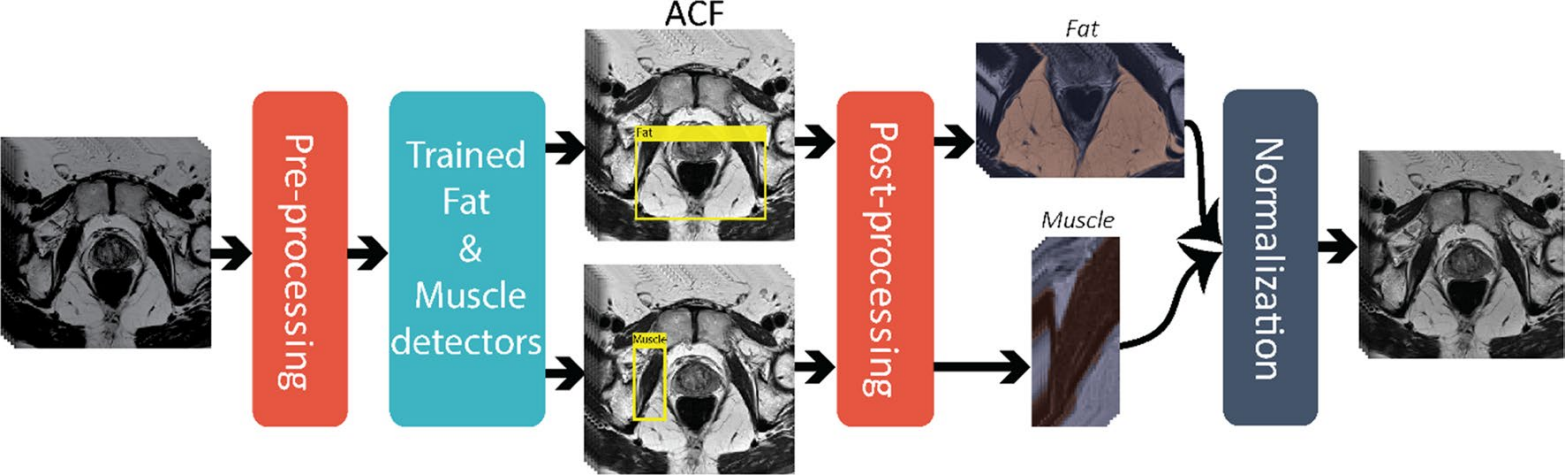
Overview of AutoRef, the proposed normalization method. The T2W images were first pre-processed including bias field correction, rescaling and resizing. Rectangles containing fat/muscle were then detected slice by slice using trained aggregate channel features (ACF) detectors. The three slices containing rectangular regions with the highest probability of containing fat/muscle were identified and post-processed by Otsu thresholding and morphological opening to extract the largest connected fat/muscle region-of-interest (ROI). From these ROIs, fat/muscle reference intensities were obtained for normalization of the 3D image intensities

Figure 1 gives an overview of the proposed method, termed AutoRef. The method contains several tuneable parameters, which were optimized as described in the next section. In the final, optimized version, the 3D T2W images were first pre-processed, which included N4 bias field correction [29], rescaling to the 99th percentile intensity value and resizing the transverse slices to 384 × 384 pixels with 0.5 × 0.5 mm in-plane resolution. Two separate aggregate channel features (ACF) object detectors [25] were then trained, using two training stages for the iterative training process, to detect rectangular ROIs containing fat and muscle (levator ani muscle) tissue on the 2D transverse slices. Both object detectors were forced to focus on regions where the ROIs were expected to minimize the detection of unwanted structures. For fat, the focus region comprised the lower (posterior) 50% of the image in the lower (inferior) 75% of the slices. For muscle, the focus region comprised the middle (posterior-anterior) 50% of the image in the middle (inferior-superior) 50% of the slices. The three slices containing the rectangular ROIs with the highest probability of fat/muscle were identified, and post-processed by Otsu thresholding [30] and morphological opening, with disk shape of one-pixel radius, to extract the largest connected bright and dark structures in the detected rectangle, representing fat and muscle ROIs, respectively. The fat ($I^{{{\text{fat}}}}$) and muscle ($I^{{{\text{muscle}}}} )$ reference intensity values were then calculated as the 90th and 10th percentiles, respectively, of the intensity values in these ROIs. Subsequently, the 3D image intensities ($I\left( {x, y, z} \right)$) were normalized to pseudo T2 values ($pT2\left( {x, y, z} \right)$) by linearly scaling $I^{{{\text{fat}}}}$ and $I^{{{\text{muscle}}}}$ to their respective T2 values at 3 T from literature ($T2^{{{\text{fat}}}}$ = 121 ms and $T2^{{{\text{muscle}}}}$ = 40 ms) [31], using Eq. (1):

1 $$pT2\left( {x, y, z} \right) = \frac{{I\left( {x, y, z} \right) - I^{{{\text{muscle}}}} }}{{I^{{{\text{fat}}}} - I^{{{\text{muscle}}}} }} \times (T2^{{{\text{fat}}}} - T2^{{{\text{muscle}}}} ) + T2^{{{\text{muscle}}}}.$$

### Training, validation and testing

The PROMISE12 dataset was shuffled and split for training (*N* = 40), validation (*N* = 20), and testing (*N* = 20) of AutoRef. Since prostate segmentations were only available for 50 cases, the splitting was semi-random and controlled in a way that ensured that only cases with the required segmentations were included in the validation and test subsets. The PROSTATEx and the in-house collected datasets were used for testing only.

The training and validation subsets were used to train the object detectors and to find the optimal pre- and post-processing settings resulting in the best performance of AutoRef. An overview of the optimization results in the validation subset is provided in Online Resource 1. The trained detectors and optimal parameter settings, as described in the previous section, were subsequently applied to normalize the images in the PROMISE12 test subset, the PROSTATEx dataset and the in-house collected dataset.

### Verification of reference tissue intensities

The reference tissue intensities extracted from muscle and fat tissue by AutoRef, $I^{{{\text{fat}}}}$ and $I^{{{\text{muscle}}}}$, respectively, were compared with those of manually drawn ROIs in the PROMISE12 test subset. In the manual approach, a researcher with 3 years of experience with prostate imaging (MRSS) delineated three ROIs in both fat and muscle tissue on what were judged to be representative T2W slices by visual inspection. The 90th and 10th percentiles of the intensity values within the manual fat and muscle ROIs, respectively, were compared to $I^{{{\text{fat}}}}$ and $I^{{{\text{muscle}}}}$ and the relative differences and absolute relative differences were calculated. Visual inspection of all automatically extracted fat and muscle ROIs from the PROMISE12 test subset, the PROSTATEx dataset, and the in-house collected dataset was performed by the same researcher to reveal any suboptimal ROIs. A ROI was considered suboptimal when it failed to detect the tissue of interest or covered additional regions not belonging to fat or muscle on any of the three slices.

### Inter- and intra-patient performance of normalization

The performance of AutoRef was compared to the original images and three other automated normalization methods, commonly used in literature, i.e. histogram stretching (Eq. (2)) [8], histogram equalization (histeq function from MATLAB^®^), and Gaussian kernel normalization (Eq. (3)) [8]:

2 $$I_{{{\text{normalized}}}} \left( {x, y, z} \right) = \frac{{I\left( {x, y, z} \right) - I_{\min } }}{{I_{\max } - I_{\min } }}$$

where $I_{\max }$ and $I_{\min }$ represent the maximum and minimum intensity values, respectively, in the original image $I$.

3 $$I_{{{\text{normalized}}}} \left( {x, y, z} \right) = \frac{{I\left( {x, y, z} \right) - \mu }}{\sigma }$$

where $\mu$ and $\sigma$ represent the mean and standard deviation of the voxel intensities in the original image $I$, respectively.

Furthermore, the performance of AutoRef using two reference tissues (as proposed) was compared to that of AutoRef^muscle^ (Eq. 4), which uses only muscle reference intensity values, as by several other studies [17, 22–24]:

4 $$pT2\left( {x, y, z} \right) = \frac{{I\left( {x, y, z} \right)}}{{I^{{{\text{muscle}}}} }} \times T2^{{{\text{muscle}}}} ,$$

where $I^{{{\text{muscle}}}}$ represents the mean value of the automatically extracted muscle ROIs and $T2^{{{\text{muscle}}}}$ the muscle T2 value from literature.

The histogram intersections (Eq. 5) of whole prostate voxel intensities of each pair of patients within the PROMISE12 test subset were used as a metric of inter-patient performance. In addition, the PROSTATEx dataset was used to separately evaluate the inter-patient histogram intersections in the peripheral (PZ) and transition zone (TZ):

5 $${\text{Intersection}}\left( {H_{x} ,H_{y} } \right) = \mathop \sum \limits_{i = 1}^{n} \min \left( {H_{x} \left( i \right),H_{y} \left( i \right)} \right)$$

where $H_{x}$ and $H_{y}$ represent the intensity histograms of patient $x$ and patient $y$, respectively, and $n$ represents the number of histogram bins (set to 100). $H_{x}$ and $H_{y}$ were normalized to the total number of voxels in the prostate or zone.

The in-house collected dataset was used to assess the intra-patient performance, by measuring the whole prostate histogram intersection between the pair of consecutive scans of the same patient (Eq. 6):

6 $${\text{Intersection}}\left( {H_{1} ,H_{2} } \right) = \mathop \sum \limits_{i = 1}^{n} \min \left( {H_{1} \left( i \right),H_{2} \left( i \right)} \right)$$

where $H_{1}$ and $H_{2}$ represent the histograms for the first and second scans of the same patient, respectively, and $n$ represents the number of histogram bins (set to 100). $H_{1}$ and $H_{1}$ were normalized to the total number of voxels in the prostate.

For all datasets, the $pT2\left( {x, y, z} \right)$ values of prostate tissue obtained with AutoRef and AutoRef^muscle^ were compared to T2 values from the literature [31]. Furthermore, the $pT2\left( {x, y, z} \right)$ values of prostate tissue obtained with AutoRef were compared between patients scanned with and without an endorectal coil.

### Classification of malignant lesions versus healthy prostate tissue

Mean intensity values were extracted from the histologically verified malignant lesions and from healthy tissue in the PZ and TZ of the PROSTATEx dataset. The values were used as predictors in logistic regression models to distinguish healthy prostate tissue from malignant lesions in the PZ and TZ, separately. To ensure representative results least influenced by how the data was split, the models were trained and tested using 10 iterations with fivefold cross-validation. In each iteration, the dataset was randomly split, in a controlled way, into training (4 folds) and testing (1 fold) datasets, allowing each fold to be used once for testing. Receiver operating characteristic (ROC) curves were created to evaluate the performance of the classifier at each iteration and the mean and 95% confidence interval (CI) of the area under the curves (AUC) was reported.

### Statistical analysis

Wilcoxon signed-rank tests were used to assess statistical differences between the manually and automatically obtained reference tissue intensities, and between the histogram intersections of the various normalization methods. Two-sample *t* tests were used to assess statistical differences between the pseudo T2 and literature T2 values of the prostate [31], and between the prostate pseudo T2 values of patients scanned with and without an endorectal coil. Wilcoxon rank-sum tests were used to assess statistical differences between the mean intensity values of healthy and malignant regions after normalization. DeLong’s method [32] was used to assess statistical differences between AUCs. The tests were followed by Benjamini–Hochberg correction for multiple comparisons [33] with false discovery rate of 0.05. Corrected *p* values less than 0.05 were considered statistically significant.

All algorithms and analyses were implemented and performed in MATLAB R2019b (The Mathworks, Nattick, MA, USA). The proposed algorithm will be made available on GitHub at https://github.com/ntnu-mr-cancer/AutoRef.

## Results

### Verification of reference tissue intensities

**Fig. 2:**
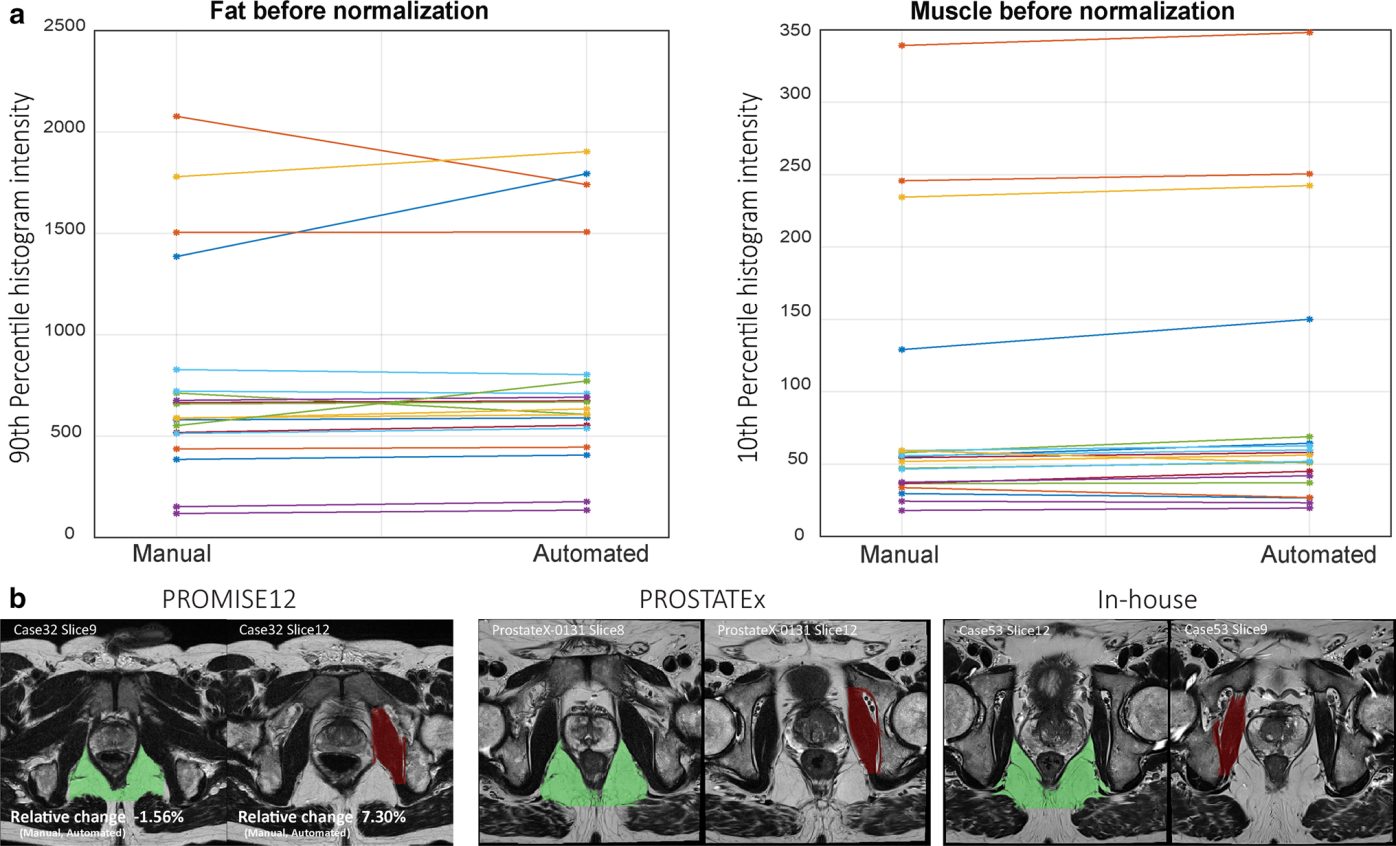
**a** The 90th and 10th percentiles of the fat and muscle intensities before normalization, respectively, in manually placed and automatically detected ROIs. **b** Representative examples of optimal fat (green) and muscle (red) ROIs automatically extracted with our method

Figure 2a shows the manually and automatically extracted fat and muscle intensities, respectively, for all cases in the PROMISE12 test subset before normalization. There were significant differences between the reference intensity values from manually and automatically detected fat (*p* = 0.048) and muscle (*p* = 0.018) ROIs, with relative differences (median (range)) of 2.52% (− 16.21 to 39.86%) for fat and 7.03% (− 20.24 to 23.20%) for muscle. The absolute relative differences [median (range)] between the manual and automated approach were 5.25% (0.17–39.86%) for fat and 9.10% (1.74–23.20%) for muscle intensities. Visual inspection revealed that automated ROIs were suboptimal in 4/20 (20%), 4/202 (2%) and 0/120 (0%) cases for fat and in 0/20 (0%), 3/202 (1.5%) and 0/120 (0%) for muscle ROIs in the PROMISE12 test subset, the PROSTATEx dataset and the in-house collected dataset, respectively, whereas the method performed well in all other cases. In the PROMISE12 test subset, 3/4 (75%) suboptimal ROIs were found in patients with an endorectal coil. Figure 2b shows representative examples of optimal ROIs automatically extracted with our method. All automatically extracted suboptimal ROIs are shown in Online Resource 2. It can be appreciated that the ‘suboptimal parts’ of the ROIs are often relatively small and of similar image intensity compared to the ‘correct parts’ of the ROIs, so their impact on the normalization is limited as shown in Online Resource 2.

### Inter- and intra-patient evaluation of normalization performance

**Fig. 3:**
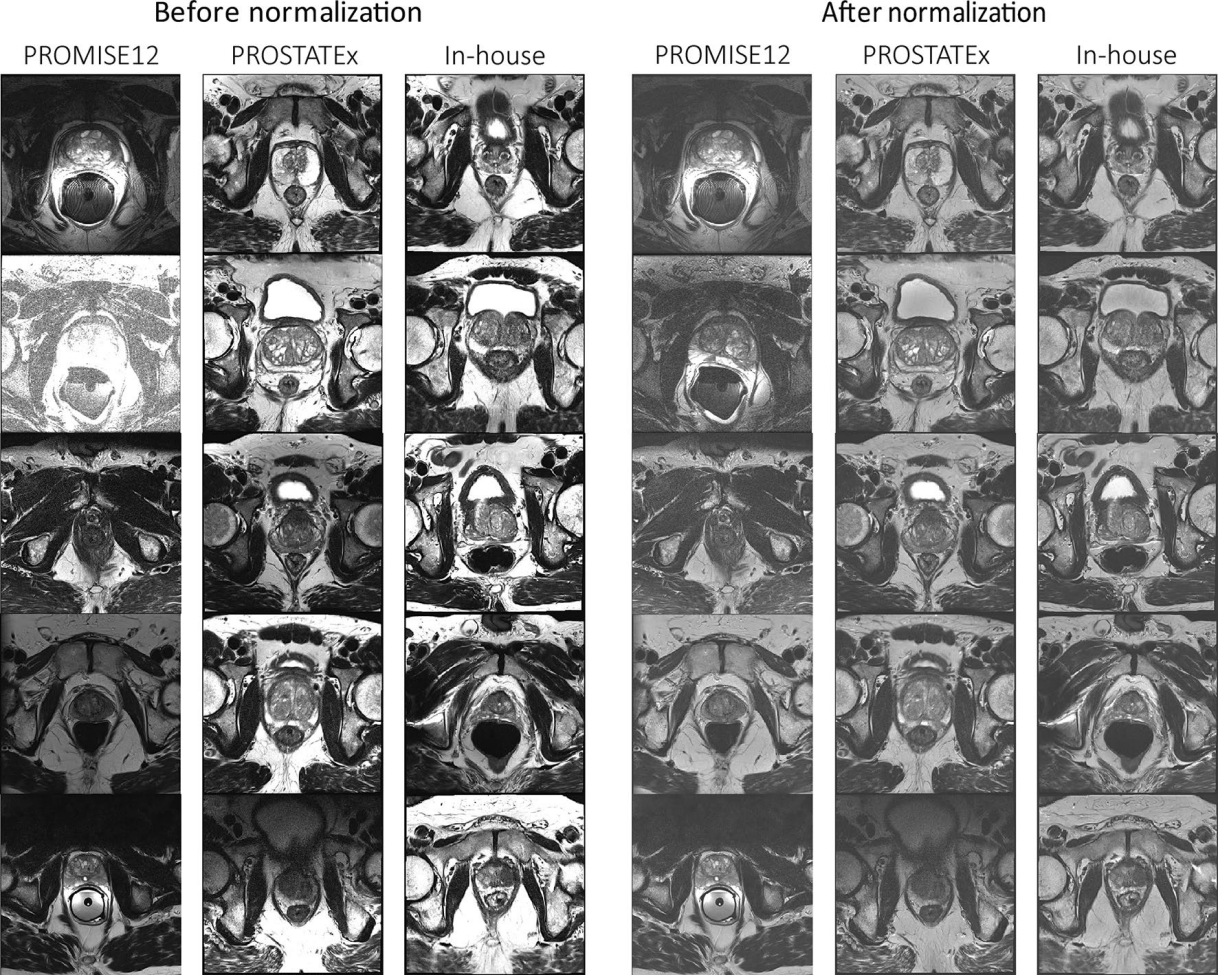
Central slice through the prostates of five patients from the PROMISE12 test subset, the PROSTATEx dataset and the in-house collected dataset before (left panel) and after normalization (right panel). In both panels, the images were window-levelled from 0 to 2 times the mean prostate intensity of all images in the respective dataset

Figure 3 shows examples from the PROMISE12 test subset, the PROSTATEx dataset, and the in-house collected dataset before and after normalization using AutoRef. The image intensities are more homogeneous within and between the datasets after normalization. This improvement is most obvious in the PROMISE12 dataset, which was acquired with varying protocols, field strengths, and at multiple centres.

**Fig. 4:**
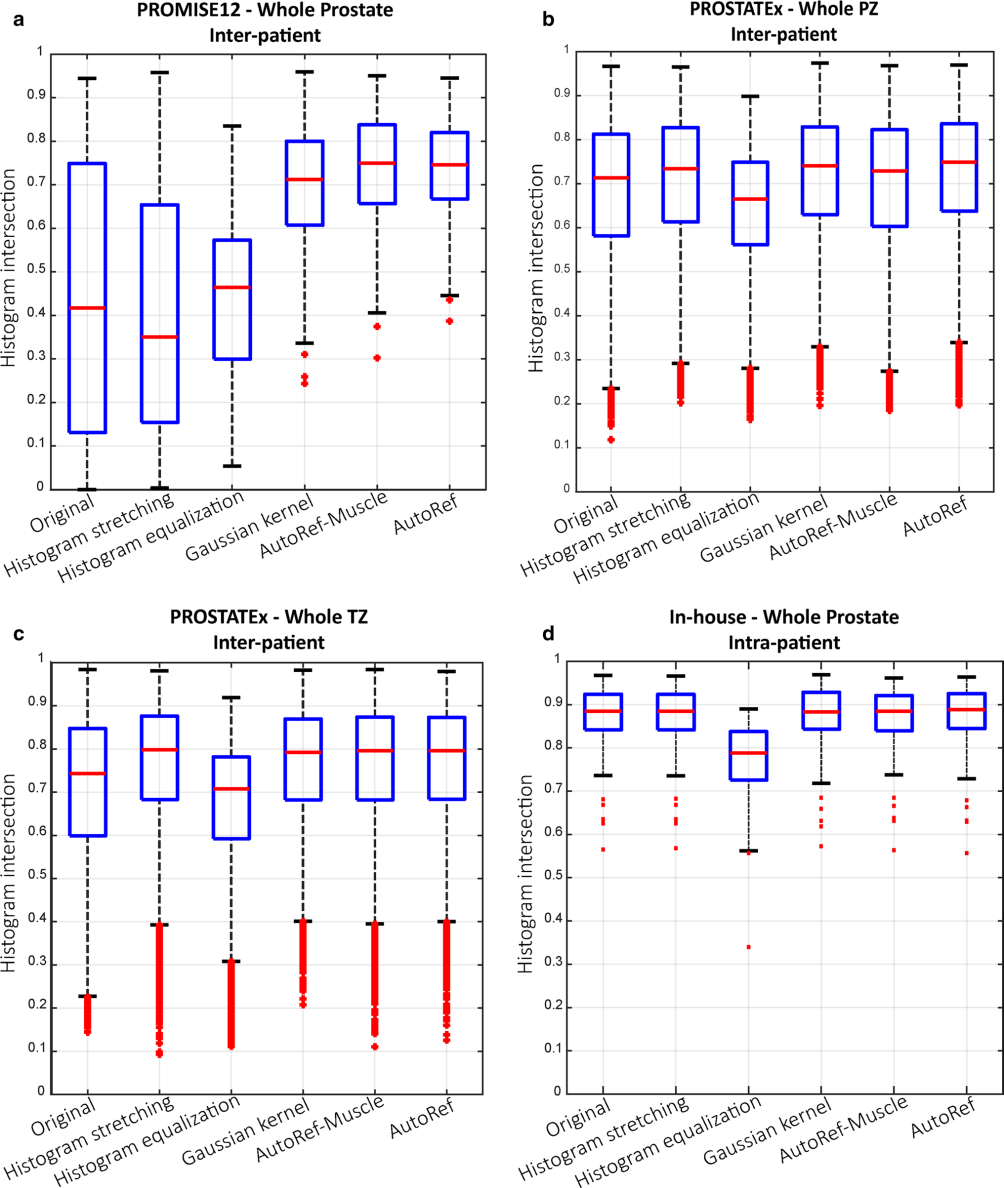
The inter-patient histogram intersections of the proposed method (AutoRef) compared to original and normalized images for the whole prostate (**a**), the peripheral (PZ; **b**) and transitional zone (TZ; **c**), respectively. The PROMISE12 test subset and PROSTATEx dataset were used in **a**, and **b** and **c**, respectively. AutoRef intersections were significantly higher (*p* < 0.001) than others, except for AutoRef^muscle^ in **a** (*p* = 0.424) and histogram stretching in **c** (*p* = 0.154). The histogram intersections between scan 1 and scan 2 of the in-house collected dataset (**d**) of AutoRef were significantly higher than for histogram equalization (*p* < 0.001), but similar to those of the other methods

The intensity histograms from the original and normalized images of PROMISE12 test subset are displayed in Online Resource 3. Figure 4a and Table 1 show that AutoRef resulted in significantly higher inter-patient intersections than the original data and the other normalization methods, except for AutoRef^muscle^.

**Table 1:** The inter-patient histogram intersections before (Original data) and after normalization with our proposed method (AutoRef) and the other investigated methods in the whole prostate, peripheral (PZ) and transition zone (TZ)

	Original data	Histogram stretching	Histogram equalization	Gaussian kernel	AutoRef^muscle^	AutoRef
Whole prostate						
Median	0.417	0.351	0.465	0.712	0.750	0.746
Range	0.000–0.945	0.003–0.958	0.054–0.835	0.244–0.960	0.302–0.951	0.387–0.945
*p* value	** < 0.001**	** < 0.001**	** < 0.001**	** < 0.001**	0.424	
PZ						
Median	0.714	0.734	0.665	0.741	0.729	0.749
Range	0.118–0.967	0.202–0.965	0.165–0.898	0.196–0.974	0.185–0.968	0.197–0.970
*p* value	** < 0.001**	** < 0.001**	** < 0.001**	** < 0.001**	** < 0.001**	
TZ						
Median	0.743	0.799	0.708	0.792	0.796	0.796
Range	0.144–0.984	0.093–0.981	0.111–0.919	0.208–0.983	0.111–0.984	0.126–0.980
*p* value	** < 0.001**	0.154	** < 0.001**	** < 0.001**	**0.003**	

The PROMISE12 test subset and PROSTATEx dataset were used in Whole prostate, and PZ and TZ, respectively. The bold values indicate a significant difference from AutoRef after correction for multiple testing

Figure 4b, c and Table 1 also present the inter-patient histogram intersections for PZ and TZ of the PROSTATEx dataset. In both zones, the histogram intersections after normalization with AutoRef were significantly higher than those of the original data and the other normalization methods, except for histogram stretching in TZ.

The intra-patient histogram intersections between scan 1 and scan 2 of the in-house collected dataset are shown in Fig. 4c and Table 2. AutoRef resulted in significantly higher intra-patient intersections than histogram equalization but performed similar to the original data and the other normalization methods.

**Table 2:** The intra-patient histogram intersections between scan 1 and scan 2 of the in-house collected dataset before (Original data) and after normalization with our proposed method (AutoRef) and the other investigated methods

	Original data	Histogram stretching	Histogram equalization	Gaussian kernel	AutoRef^muscle^	AutoRef
Median	0.884	0.885	0.788	0.883	0.884	0.889
Range	0.565–0.968	0.568–0.966	0.340–0.890	0.573–0.969	0.563–0.961	0.557–0.964
*p* value	0.640	0.640	** < 0.001**	0.774	0.640	

The bold values indicate a significant difference from AutoRef after correction for multiple testing

**Fig. 5:**
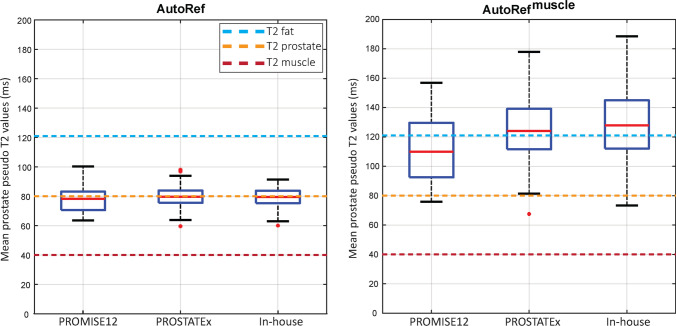
Box and whisker plots of the mean prostate pseudo T2 values of the patients in the PROMISE12 test subset, the PROSTATEx dataset and the in-house collected dataset after normalization with the proposed dual-reference normalization method (AutoRef) and single reference tissue normalization (AutoRef^muscle^). The dashed lines correspond to the T2 values reported in literature. All the mean prostate T2 values for AutoRef^muscle^, but not AutoRef, were significantly higher than those reported in literature (*p* < 0.001)

Figure 5 compares the pseudo T2 values of the whole prostate obtained with AutoRef and AutoRef^muscle^ with those reported in the literature (80 ± 34 ms) [31]. Using AutoRef, the mean ± standard deviation prostate pseudo T2 values were 78.49 ± 9.42 ms (*p* = 0.063), 79.69 ± 6.34 ms (*p* = 0.486) and 79.29 ± 6.30 ms (*p* = 0.161) for PROMISE12 test subset, the PROSTATEx dataset and the in-house collected dataset, respectively. Pseudo T2 values were not significantly different between patients scanned with (83.15 ± 8.85 ms) or without (79.36 ± 6.41 ms) an endorectal coil (*p* = 0.690). Using AutoRef^muscle^, the prostate pseudo T2 values were significantly higher (*p* < 0.001) than literature values for all the datasets.

### Classification of malignant lesions versus healthy prostate tissue

**Fig. 6:**
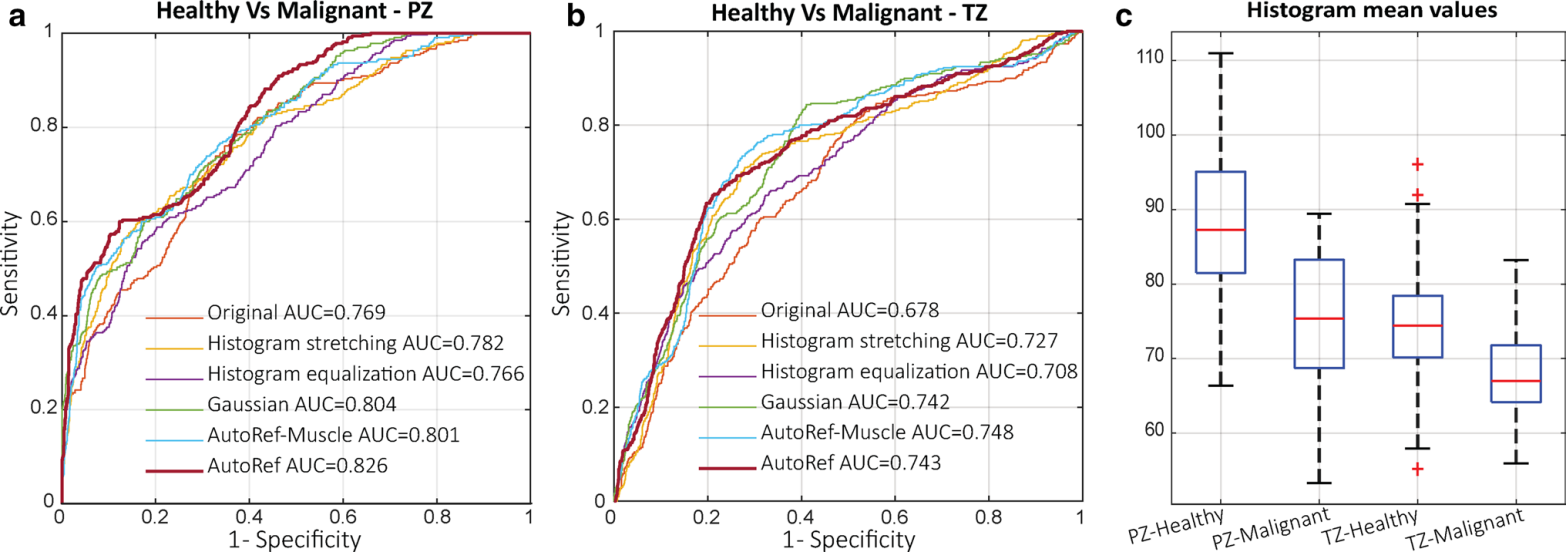
The receiver operating characteristic curves and areas under the curves (AUC; mean of 10 iterations) for the proposed method (AutoRef), the original images and the other investigated normalization methods in the peripheral (PZ; **a**) and transitional zone (TZ; **b**). In PZ, the AUC for AutoRef was significantly higher than that of the other methods (*p* < 0.001), whereas in TZ it was significantly higher than the original data (*p* < 0.001), histogram stretching (*p* = 0.010) and histogram equalization (*p* = 0.007). The mean pseudo T2 values (**c**) were significantly different between healthy and malignant regions in both the PZ and TZ. (*p* < 0.001)

Figure 6a, b and Table 3 compare the performances (ROC curves and mean AUCs of the 10 iterations, respectively) of AutoRef and other methods in the classification of healthy tissue versus biopsy-confirmed cancer regions. In the PZ, AutoRef performed significantly better than the original data and the other normalization methods. In the TZ, the performance was similar to Gaussian kernel normalization and AutoRef^muscle^, but significantly better than the original data, histogram stretching and histogram equalization. Figure 6c shows box and whisker plots of the mean pseudo T2 values of healthy and malignant regions after AutoRef normalization, which were significantly different in both the PZ and TZ (*p* < 0.001).

**Table 3:** Areas under the curves (AUC; mean of 10 iterations) for the proposed method (AutoRef), the original images and the other investigated normalization methods when classifying healthy versus malignant tissues in the peripheral (PZ) and transition zone (TZ)

	Original data	Histogram stretching	Histogram equalization	Gaussian kernel	AutoRef^muscle^	AutoRef
PZ						
AUC	0.769	0.782	0.766	0.804	0.801	0.826
95% CI	0.765–0.772	0.778–0.787	0.761–0.771	0.800–0.808	0.797–0.805	0.822–0.830
*p* value	** < 0.001**	** < 0.001**	** < 0.001**	** < 0.001**	** < 0.001**	
TZ						
AUC	0.678	0.727	0.708	0.742	0.748	0.743
95% CI	0.672–0.684	0.723–0.730	0.703–0.712	0.739–0.746	0.744–0.751	0.738–0.748
*p* value	** < 0.001**	**0.010**	**0.007**	0.881	0.559	

The bold values indicate a significant difference from AutoRef after correction for multiple testing

*CI* confidence interval

## Discussion

In this paper, we propose a new method for automated dual-reference tissue normalization of T2W images of the prostate, which shows promise for quantitative assessment of prostate cancer and could ease the comparison of T2-weighted images between and within patients. The proposed method successfully uses a simple object detector to extract reference tissue intensities from fat and muscle surrounding the prostate, which are subsequently used for intensity normalization of the 3D T2-weighted image. The proposed method generally resulted in higher inter-patient histogram intersections compared to the other investigated automated normalization methods, which indicates that the normalized intensity values in the prostate are more similar between images. Furthermore, the proposed method resulted in images with pseudo T2 values comparable to T2 values reported in the literature [31]. Lastly, as demonstrated by the improved classification of healthy versus malignant tissue, the proposed method successfully reduced the inter-patient variation in T2W image intensities, which could facilitate the extraction and application of meaningful intensity-based image features for quantitative assessment of prostate cancer, e.g. in a radiomics or computer-assisted diagnosis framework [34].

T2W normalization is paramount for the quantitative assessment of prostate cancer, and several methods have been previously proposed in the literature. Liu et al. [13] defined a non-parametric normalization standard as the median image intensity plus two times the inter-quartile range. Artan et al. [14] and Ozer et al. [15] normalized T2W images in a way similar to the Gaussian kernel method investigated here, but with the mean and standard deviation extracted from the PZ instead of the entire image. However, these methods require manual delineation of the PZ and might not be valid if the image intensities do not follow a Gaussian distribution [10]. Lemaitre et al. [10] chose to normalize the images using a parametric model assuming a Rician distribution of the voxel intensities in the whole prostate. Yet differently, Nyúl et al. [7] proposed a two-stage method, wherein the first stage a template histogram with landmarks of interest is created and in the second stage new histograms are mapped via linear transformation to the template. This method assumes that the MR images of the same sequence should have the same intensity distribution, which might not be the case for varying protocols. Vos et al. proposed a sequence-based approach, which depends on the original T2W signal, proton density value, a reference tissue, and a known sequence model to estimate new normalized T2W images [35]. Although this approach performs well, the intricate nature and additional scan time make its practical implementation difficult. Niaf et al. [20, 21] investigated a single reference tissue method that normalizes the image intensities by dividing by the mean intensity value of the bladder. Likewise, Peng et al. [17] normalized the images separately using each of the levator ani muscle, urinary bladder, and pubic bone, and concluded that using levator ani muscle as a single reference tissue gave the best results. In this work, the performance of AutoRef using only muscle reference intensities was shown to be generally inferior to that based on a dual-reference tissue normalization approach, and unable to correctly map the image intensities to literature T2 values. Our method uses fat as a second reference tissue because it typically has high T2W intensity values, thus together with muscle covering the full range of expected prostate intensity values, it is present in all images and less vulnerable to external factors than for example the urinary bladder.

Recently, Stoilescu et al. [19] showed that multi-reference tissue normalization of T2W prostate images significantly improved prostate cancer classification accuracy in comparison to non-normalized images. Four reference tissues were used based on manually annotated ROIs, which currently hinders the implementation of the method in clinical practice. Therefore, in our work, we developed an automated approach for detecting ROIs to enable multi-reference tissue normalization using two reference tissues (fat and levator ani muscle). The ACF detector used in this work is a relatively simple, classical machine learning approach that was able to accurately detect the fat and muscle ROIs in nearly all cases, despite the small training dataset (*N* = 40). Exceptions were found in 8/342 (2%) cases for fat and 3/342 (1%) cases for muscle ROIs when considering all patients, and in 1/331 (0.3%) and 3/331 (1%) cases, respectively, when considering patients scanned without an endorectal coil. The detection of fat thus performs worse in patients scanned with an endorectal coil but this may not pose a problem in clinical practice, as 3 T MRI with body surface coils is currently the recommended and preferred method. The detection of both fat and muscle may be further improved by using a larger dataset for training, while the method can also be extended to include more reference tissues if deemed necessary, which is subject of further investigation.

Although AutoRef generally performed better than or similar to the other normalization methods in all datasets, the largest differences were observed in the multi-centre, multi-vendor PROMISE12 dataset. In this dataset, images were acquired with 1.5 T or 3 T scanners, with or without an endorectal coil, and with different acquisition protocols, all of which are likely to influence the T2W image intensity. An important advantage of our method to the other investigated normalization methods is that the image intensities could be correctly mapped to literature T2 values [31], irrespective of these factors. The pseudo T2 values could be an interesting alternative to quantitative T2 mapping, given the limited scan time available in clinical practice, but this needs further investigation in studies where T2 maps are also acquired. However, it should be noted that AutoRef does not correct for local differences in signal intensities caused by the non-uniform sensitivity of the receiver coils. This effect is especially apparent for images acquired with an endorectal coil, which typically shows an intensity profile inversely related to proximity to the coil. Although we showed that the mean pseudo T2 values of images acquired with an endorectal coil were comparable to those acquired with body surface coils, there may be differences in intensity distribution within the prostate gland that are not accounted for by AutoRef.

In the intra-patient evaluation, AutoRef had similar intra-patient histogram intersections compared to the original data and most of the other investigated methods. This probably reflects the limited variability in the in-house collected dataset, which has been acquired at the same centre, the same scanner, with the same protocols at a relatively short interval between scans. It would be insightful to assess the performance of the method in a dataset where the same patients are systematically scanned at different hospitals, but such data are probably scarce.

Normalization with the proposed method resulted in a significantly higher AUC for the classification of histologically verified PZ lesions compared to the other methods. For TZ lesions, the AUC was significantly higher than the original data, histogram stretching and histogram equalization, and on par with the other normalization methods. However, the differences in the classification performance were relatively small, which again may be the result of the limited variability in a dataset acquired at a single centre and with a single protocol [28]. Furthermore, considerable overlap in pseudo T2 values was still present between healthy tissue and malignant lesions, especially in the TZ, indicating that pseudo T2 values alone may not be sufficient to detect prostate cancer in clinical practice.

Our study has some limitations. Quantitative T2 maps were not available for the patients included in this study, which hindered a direct comparison of the pseudo T2 values with a gold standard. Although we included several commonly applied automated normalization methods in this study, there are still many more described in the literature, as discussed above, that may perform better than those included here. In addition, it would be interesting to compare the performance of the proposed object detector to that of semantic segmentation for detecting ROIs, which will be subject to further research. Despite these limitations, we have shown that our proposed method for automated dual-reference tissue normalization performed equal to or better than other automated normalization methods. The method requires no manual input and the resulting images can be used for both quantitative and qualitative assessment of prostate cancer.

## Conclusion

We successfully developed a method for automated dual-reference tissue normalization of T2W MR images of the prostate using object recognition. The method was shown to reduce T2W intensity variation between scans and could improve the quantitative assessment of prostate cancer on MRI.

## Electronic supplementary material

Below is the link to the electronic supplementary material.

Supplementary file1 (PDF 160 kb)

Supplementary file2 (PDF 1280 kb)

Supplementary file3 (PDF 266 kb)
