# Supplementary material for: Automated reference tissue normalization of T2-weighted MR images of the prostate using object recognition
Source: MAGMA. 2020 Jul 31;34(2):309–21. doi: 10.1007/s10334-020-00871-3 (PMC8018925; doi:10.1007/s10334-020-00871-3)
Supplement: Supplementary file 1 — Supplementary file1 (PDF 160 kb) [file 10334_2020_871_MOESM1_ESM.pdf]

Magnetic Resonance Materials in Physics, Biology and Medicine

Automated reference tissue normalization of T2-weighted MR images of the prostate using object recognition

Mohammed R. S. Sunoqrot, Gabriel A. Nketiah, Kirsten M. Selnæs, Tone F. Bathen and Mattijs Elschot

Online Resource 1

Optimization of the AutoRef pre- and post-processing settings on the validation set

We optimized the following pre- and post-processing parameters on the validation set: type of scaling, number of the detector training stages, radius of the morphological opening structure, number of the evaluated slices, low (muscle) and high (fat) percentiles used to calculate the reference intensity values and focus regions (inferior-superior slices and posterior-anterior rows) for fat and muscle. The table below lists the effect on the median histogram intersection (HI) when changing one parameter at the time, while keeping the other parameters set at the ***bold*** *values. The* ***bold*** *set of parameters was considered our optimal set as no further improvements of more than 1% could be achieved by additional tuning of the parameters (i.e. Change in HI median < 1% for all parameter values).*

Settings	HI median	Change in HI median (%)	Selected as an optimal
		Scaling	
**99th Percentile**	0.76997959	**0**	***X***
Median	0.77183996	0.241613039	
Max	0.76662009	-0.436310938	
None	0.75950878	-1.359881374	
		Number of detector Stages	
1	0.77192269	0.252357431	
**2**	0.76997959	**0**	***X***
3	0.76997959	0	
4	0.76997959	0	
5	0.76997959	0	
		Morphological opening structure radius	
**1**	0.76997959	**0**	***X***
2	0.77002793	0.006277583	
3	0.7677967	-0.283500407	
4	0.76807893	-0.246846188	
5	0.7675968	-0.309461357	
		Number of evaluated Slices	
1	0.76806568	-0.248566275	
2	0.77272643	0.356741256	
**3**	0.76997959	**0**	***X***
4	0.76859372	-0.179988324	
5	0.77103737	0.137376853	

Low percentile (Muscle)			
25	0.7736801	0.48059794	
20	0.77221788	0.290693982	
15	0.77017365	0.025203469	
**10**	0.76997959	**0**	***X***
5	0.76839093	-0.206325746	
High percentile (Fat)			
75	0.76036739	-1.044199148	
80	0.76423774	-0.540504432	
85	0.76676418	-0.211708731	
**90**	0.76839093	**0**	***X***
95	0.76178638	-0.859529925	
Focus Region Slices (Fat)			
All [0-100%]	0.76997959	0	
Lower Half [0-50%]	0.76997959	0	
**lower 75% [0-75%]**	0.76997959	**0**	***X***
Middle [25%-75%]	0.76964891	-0.042946643	
Upper Half [50%-100%]	0.74567085	-3.157063397	
Upper 75% [25%-100%]	0.76964891	-0.042946643	
Focus Region Rows (Fat)			
All [0-100%]	0.76968231	-0.03860894	
**Lower Half [0-50%]**	0.76997959	**0**	***X***
lower 75% [0-75%]	0.76968231	-0.03860894	
Middle [25%-75%]	0.75187963	-2.3507062	
Upper 75% [25%-100%]	0.75562408	-1.864402074	
Focus Region Slices (Muscle)			
All [0-100%]	0.77053318	0.071896978	
Lower Half [0-50%]	0.7737537	0.490156996	
lower 75% [0-75%]	0.76997959	0	
**Middle [25%-75%]**	0.76997959	**0**	***X***
Upper Half [50%-100%]	0.7679594	-0.262369737	
Upper 75% [25%-100%]	0.77053318	0.071896978	
Focus Region Rows (Muscle)			
All [0-100%]	0.76913026	-0.110305212	
Lower Half [0-50%]	0.76709563	-0.374550451	
lower 75% [0-75%]	0.76913026	-0.110305212	
**Middle [25%-75%]**	0.76997959	**0**	***X***
Upper 75% [25%-100%]	0.76912854	-0.110528935	
