# Supplementary material for: Automated reference tissue normalization of T2-weighted MR images of the prostate using object recognition
Source: MAGMA. 2020 Jul 31;34(2):309–21. doi: 10.1007/s10334-020-00871-3 (PMC8018925; doi:10.1007/s10334-020-00871-3)
Supplement: Supplementary file 2 — Supplementary file2 (PDF 1280 kb) [file 10334_2020_871_MOESM2_ESM.pdf]

Magnetic Resonance Materials in Physics, Biology and Medicine

Automated reference tissue normalization of T2-weighted MR images of the prostate using object recognition

Mohammed R. S. Sunoqrot, Gabriel A. Nketiah, Kirsten M. Selnæs, Tone F. Bathen and Mattijs Elschot

Online Resource 2

All suboptimal automatically extracted ROIs using AutoRef and their impact on the normalization

The suboptimal ROIs impact on the normalization:

To measure the suboptimal ROIs impact on the normalization, we compared the medians of the histogram intersections of all the cases with suboptimal ROIs with an equivalent number (overall and per dataset) of randomly selected cases with optimal ROIs. For each case, the median of the histogram intersections with the rest of its dataset cases, excluding those with suboptimal ROIs, was taken. Wilcoxon signed rank test was used to assess the statistical difference and p-values less than 0.05 were considered statistically significant. The test showed no significant difference between the medians (p=0.278).

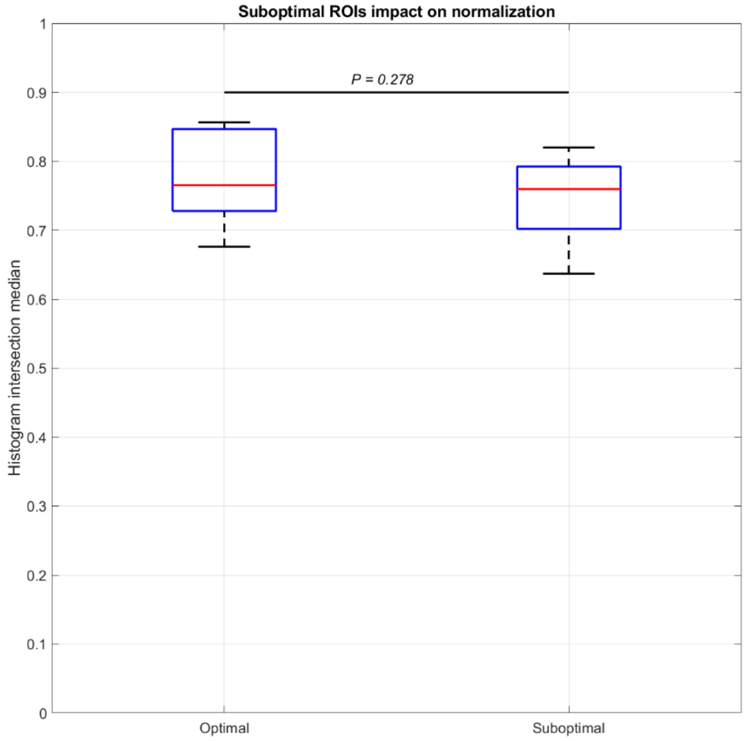

The suboptimal automatically extracted ROIs using AutoRef:

Below, each row represents a case, while the columns are the 3 detected regions-of-interest (ROIs) for that case. The fat ROIs are in green and the muscle ROIs are in red. Under each case of PROMISE12 test subset, the absolute relative difference of the reference intensity values between the manual and automated approach has been given.

Criteria:

A case was considered a suboptimal when any of its extracted ROIs failed to detect the fat or muscle tissue, or covered additional regions not belonging to fat or muscle.

PROMISE12 test subset

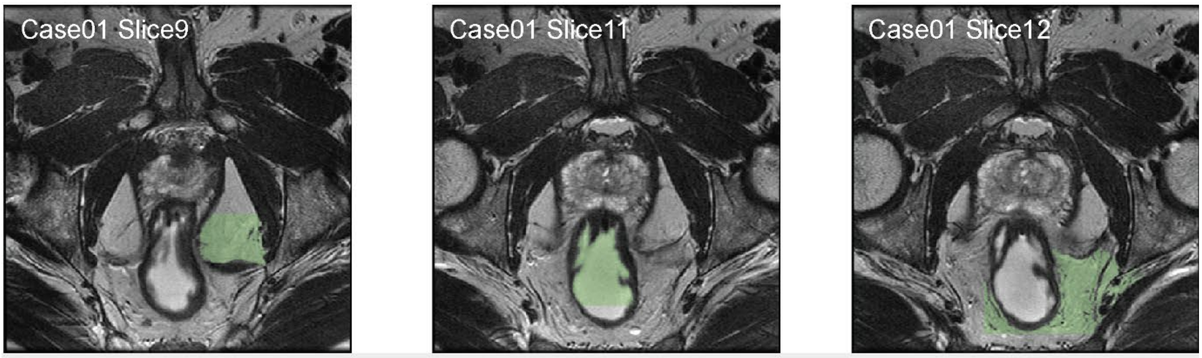

The absolute relative difference = 16.37%

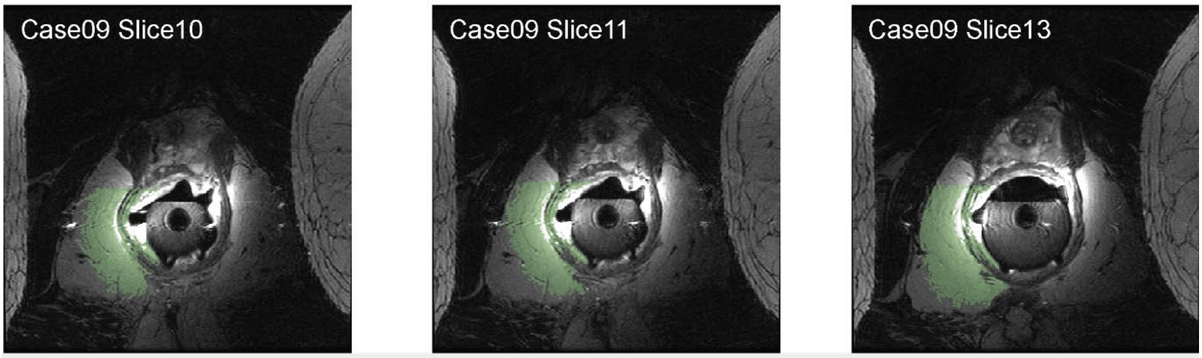

The absolute relative difference = 29.46%

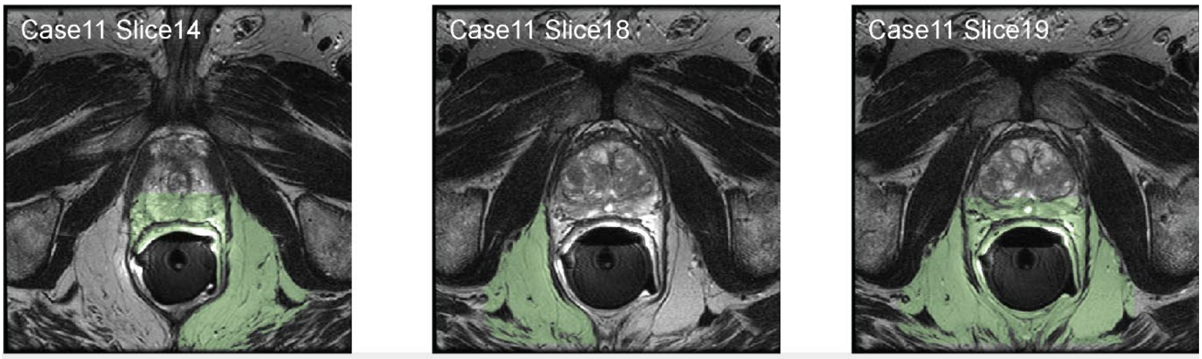

The absolute relative difference = 6.96%

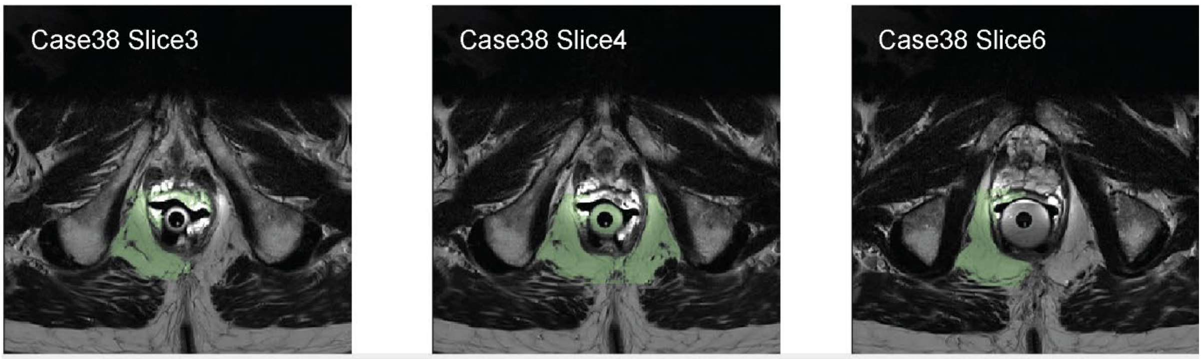

The absolute relative difference = 39.86%

PROSTATEx dataset

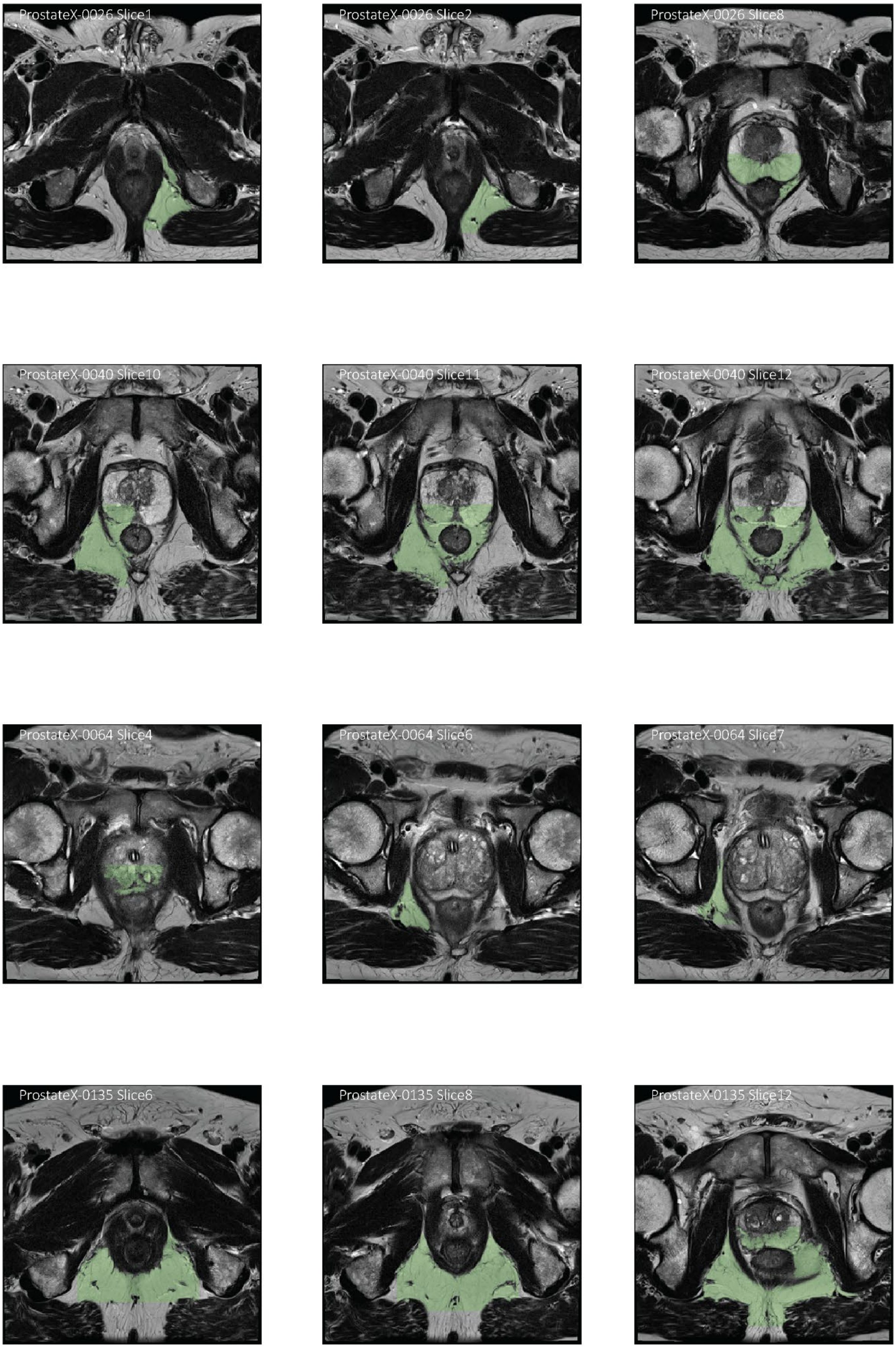

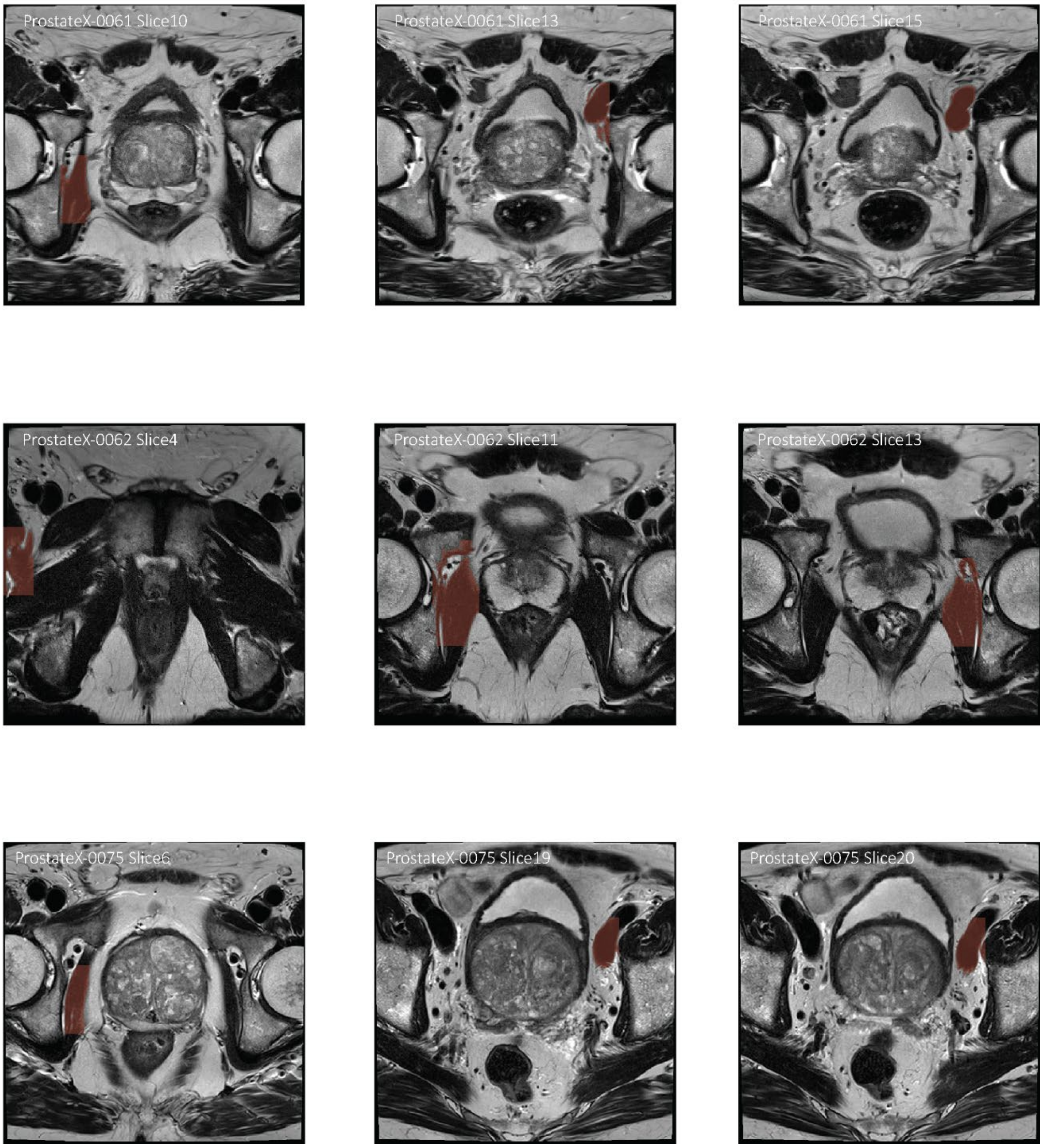
ProstateX-0061 Slice10
ProstateX-0061 Slice13
ProstateX-0061 Slice15
ProstateX-0062 Slice4
ProstateX-0062 Slice11
ProstateX-0062 Slice13
ProstateX-0075 Slice6
ProstateX-0075 Slice19
ProstateX-0075 Slice20
