# Supplementary material for: Automated reference tissue normalization of T2-weighted MR images of the prostate using object recognition
Source: MAGMA. 2020 Jul 31;34(2):309–21. doi: 10.1007/s10334-020-00871-3 (PMC8018925; doi:10.1007/s10334-020-00871-3)

Magnetic Resonance Materials in Physics, Biology and Medicine

Automated reference tissue normalization of T2-weighted MR images of the prostate using object recognition

Mohammed R. S. Sunoqrot, Gabriel A. Nketiah, Kirsten M. Selnæs, Tone F. Bathen and Mattijs Elschot

Online Resource 3

A visual representation of the image intensity histograms from the original and normalized images of PROMISE12 test subset. AutoRef is our proposed method

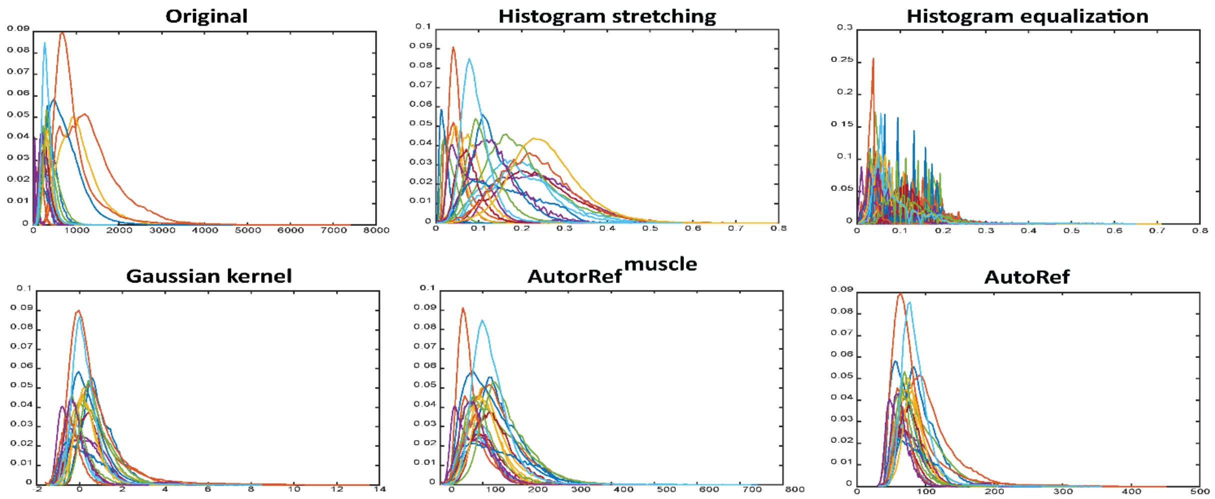

Supplement: Supplementary file 3 — Supplementary file3 (PDF 266 kb) [file 10334_2020_871_MOESM3_ESM.pdf]
